# Effect of measurement procedure errors on assessing lung fluid via remote dielectric sensing system

**DOI:** 10.1038/s41598-024-65074-5

**Published:** 2024-06-18

**Authors:** Wei-Ting Chen, Yi-Ju Tsai, Hsiao-Chen Chou, Yi-Chih Pu, Jung-Yien Chien, Chun-Ta Huang

**Affiliations:** 1 Department of Anesthesiology, Shin Kong Wu Ho-Su Memorial Hospital, Taipei, Taiwan; 2 School of Medicine, College of Medicine, Fu Jen Catholic University, New Taipei City, Taiwan; 3 Department of Internal Medicine, National Taiwan University Hospital, No. 7, Chung-Shan South Road, Taipei, Taiwan

**Keywords:** Heart failure, Intra-rater reliability, Lung fluid content, Remote dielectric sensing system, Standardized operating procedure, Sensors and probes, Cardiology, Imaging techniques

## Abstract

The study assessed the impact of procedural errors on the remote dielectric sensing system (ReDS), a non-invasive lung fluid assessment technology, in an Asian cohort. Healthy volunteers underwent ReDS measurements following manufacturer’s instructions, with two consecutive measurements one minute apart. A subset of 20 participants had modified procedure settings. Reliability was measured using intraclass correlation coefficient (ICC). The study included 86 healthy volunteers, and all ReDS measurements fell within the recommended normal range. The intra-rater reliability of ReDS measurements was excellent, with an ICC of 0.968. Among the subset of 20 subjects, deviations in height and weight did not significantly affect ReDS values. However, deviations in chest size by ± 3 cm had a noticeable impact on ReDS measures, and incorrect station selection led to fluctuations in ReDS readings. In conclusion, the ReDS system demonstrated excellent intra-rater reliability and applicability in an Asian cohort. Procedural errors, such as chest size measurement and station selection, significantly influenced ReDS measurements. Adherence to standardized operating procedures is crucial to ensure accurate and consistent results. These findings highlight the importance of adherence to manufacturer instructions when utilizing ReDS for lung fluid assessment, thereby enhancing its reliability and clinical applicability.

## Introduction

The remote dielectric sensing system (ReDS) is an FDA and CE approved non-invasive technology developed by Sensible Medical Innovations, based in Netanya, Israel. It utilizes electromagnetic energy to rapidly assess lung fluid content in absolute values. The fluid content parameter is expressed as a percentage (%), representing the volume of fluid within the lung relative to the total lung volume. Primarily designed for lung fluid monitoring, the ReDS system underwent initial testing in patients with heart failure, revealing a nearly linear relationship between ReDS measurements and CT fluid content^1^. Furthermore, a robust correlation was observed between reductions in ReDS readings and changes in net fluid balance^1^. Subsequent studies have confirmed the accuracy of its quantification of lung fluid content in comparison to CT scans in patients with and without heart failure^2^, and also demonstrated a strong correlation between ReDS measures and invasively determined pulmonary artery wedge pressure among patients with heart failure^3^. Clinical observations and trials have provided compelling evidence supporting the benefits of utilizing ReDS measurements in guiding heart failure management^4–7^. Additionally, recent research has indicated that the ReDS system could be valuable in identifying acute heart failure in patients who presented to the emergency department with dyspnea^8^. The first published meta-analysis, involving 985 patients across 7 studies, showed a 60% reduction in heart failure patient rehospitalization rates when using ReDS monitoring compared to those without^9^.

While the ReDS system is acknowledged for its ability to provide reproducible information regarding lung fluid content, it is crucial to adhere to the manufacturer’s instructions when taking measurements. In a pioneer study by Hori et al.^10^, the inter-rater and intra-rater reliability of ReDS measurements were examined. The results revealed a high level of intra-rater reliability, with intra-class correlation coefficients ranging from 0.966 to 0.988^10^. However, it is worth noting that the inter-rater reliability exhibited a lower intra-class correlation coefficient of 0.683^10^. The reason for the less than perfect inter-rater reliability remains undetermined.

There are several important instructions to ensure accurate and reliable ReDS measurements. First, it is recommended to have the patient wear light clothing during the measurement. Second, it is crucial to ensure that all patient details are correctly entered into the system. Last, the sensor unit should be applied properly on the patient for optimal results. However, the extent to which errors made during the operating procedure can affect the results of ReDS measurements is still unknown. In this study, therefore, our aim was to investigate the impact of procedure details on the measurements of the ReDS system. Specifically, we examined factors such as incorrect height or weight entry, misplacement of the back sensor station, and deviations in chest size measurement.

## Methods

### Study settings and subjects

This prospective observational study was conducted at the National Taiwan University Hospital in Taiwan. Between July 2022 and May 2023, participants were invited to join the study if they met the following criteria: they were aged 20 years or older, had no history of medical illnesses, and provided written informed consent. Exclusion criteria for this study included the presence of a rib fracture, flail chest, congenital anomaly of the thoracic cavity, or significant kyphoscoliosis; height below 155 cm or above 195 cm; and a body mass index (BMI) less than 22 or greater than 36 kg/m^2^. The study was conducted in compliance with the Declaration of Helsinki, and the protocol received approval (202104093DIPA) from the Research Ethics Committee of the National Taiwan University Hospital. Before the study procedures began, all participants provided written informed consent.

### ReDS system

This study utilized the ReDS Pro System (Sensible Medical Innovations), which employs a technology that has been previously described^1^. To briefly explain the technology, two sensors are placed on the right anterior and posterior thoracic wall, without the need for direct skin contact. Electromagnetic signals are emitted through the right thorax and captured after passing through the tissues, providing information about their combined dielectric properties. Each tissue possesses distinct dielectric coefficients, with water having a high coefficient and air having the lowest. In the lung tissues, their dielectric coefficient is primarily influenced by their fluid content. The ReDS measurement itself takes only 45 s, and the entire procedure can be completed within 5 min by skilled practitioners. The established range for normal ReDS values indicating lung fluid content is between 20 and 35%, as determined previously^2^. According to the manufacturer’s user manual, the display range for the fluid content parameter on the device is 15–60%. A recent paper provided more detailed description of the ReDS device and its operating instructions^11^.

### Study protocol

All ReDS measurements were conducted following manufacturer’s instructions. The measurements were performed while the subjects were seated on a long back chair, and a single operator who was certified by the manufacturer carried out the measurements. The study protocol consisted of two parts. In the first part, two consecutive measurements were taken on each participant, with a one-minute interval between them. The readings obtained were then averaged for further analysis. This part of the protocol aimed to assess the performance of the ReDS system in an Asian cohort and determine the intra-rater reliability of the ReDS measures.

**Figure 1:**
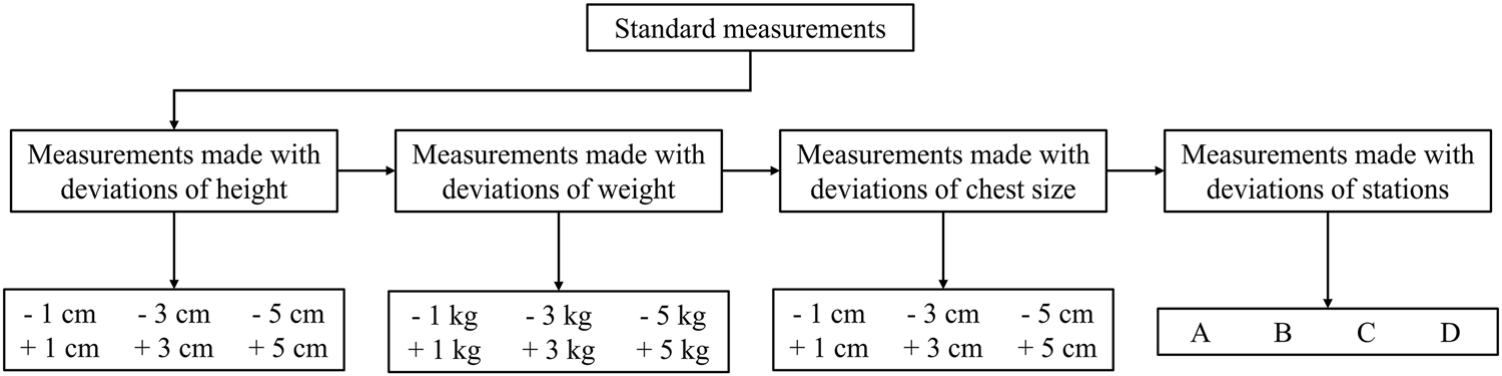
Study protocol.

In the second part of the study, a subset of 20 subjects was randomly chosen from the cohort. The aim of this part was to assess the impact of procedural errors on the ReDS readings. The subjects underwent serial ReDS measurements with specific modifications to the procedure settings (Fig. 1). In each specified procedure setting, measurements were taken twice with a 1 min interval, and the values were averaged for statistical analysis. Initially, after the standard measurements, ReDS readings were obtained while manipulating the height entry by ± 1 cm, 3 cm, or 5 cm from the true value. Subsequently, ReDS measurements were taken with modifications to the weight entry by ± 1 kg, 3 kg, or 5 kg from the actual value. We then obtained ReDS values after modifying the chest size measurement by ± 1 cm, 3 cm, or 5 cm from the measured value. Finally, ReDS measurements were conducted with the selector set to stations other than the one recommended by the system.

### Sample size calculation

The test–retest reliability of ReDS measurement, assessed using the intraclass correlation coefficient (ICC), was expected to be 0.95^10^. The measurement was taken with two repetitions. The minimum acceptable ICC was set at 0.9. For a two-tailed test, a significance level of 0.05 and a power of 90% were chosen. Anticipating no dropout rate, a sample size of at least 83 subjects was required for this study^12^.

### Statistical analysis

The numerical variables were reported as means ± standard deviations (range), while categorical variables were presented as numbers and percentages. For between-group comparisons, the paired t-test was employed. Intra-rater reliability was assessed using an ICC. The ICC is a numeric value ranging between 0 and 1. Values below 0.5 are indicative of poor reliability, values between 0.5 and 0.75 suggest moderate reliability, values between 0.75 and 0.9 indicate good reliability, and any value above 0.9 signifies excellent reliability^13^. The analyses were conducted using SPSS Statistics 20.0 software (IBM Corp, Armonk, NY, US). Statistical significance was determined using two-tailed *P* values < 0.05.

## Results

### ReDS in an Asian cohort

During the study period, a total of 86 healthy volunteers (Table 1) were included in the study. The average age of the study cohort was 41 years, with approximately 40% of them being male. The average BMI was 24 kg/m^2^, and the average height and weight were 165 cm and 65 kg, respectively. In our cohort, the 172 ReDS measurements, with two measurements for each subject, ranged from 20 to 34%, which falls within the normal range recommended by the manufacturer.

**Table 1:** Baseline subject features.

Characteristic	Data
Patient no	N = 86
Age, years	41 ± 12 (24–71)
Male sex	34 (40)
Height, cm	165 ± 7 (155–180)
Weight, kg	65 ± 13 (40–110)
Body mass index, kg/m^2^	24 ± 4 (22–36)
ReDS, first measurement, %	26 ± 3 (20–33)
ReDS, second measurement, %	27 ± 3 (20–34)
ReDS, average, %	26 ± 3 (20–33)

*ReDS* remote dielectric sensing.

### Intra-rater reliability

**Figure 2:**
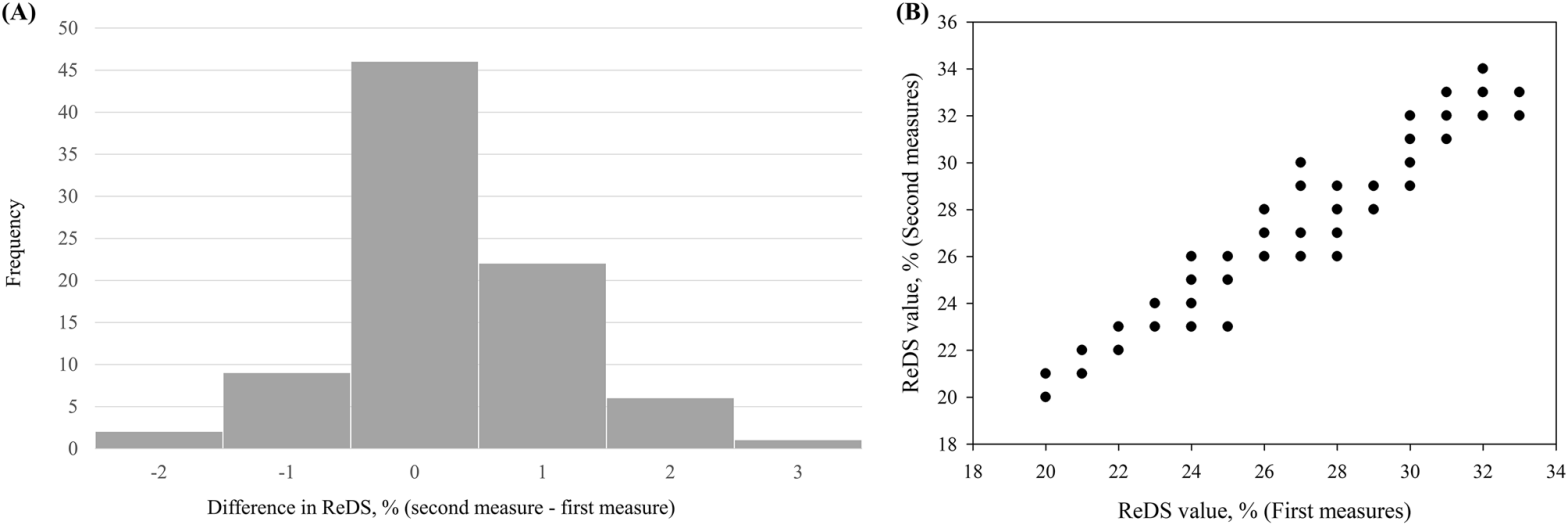
Changes in remote dielectric sensing (ReDS) values between the first and second measurements, as displayed in the histogram (**A**) and scatterplot (**B**).

The average ReDS readings (Table 1) for the first and second measurements in the study cohort were 26% and 27%, respectively. Figure 2 provides a detailed visualization of the changes in ReDS values between the first and second measurements. The ICC between the two measures was 0.968 (95% CI 0.951–0.979), indicating excellent reliability.

### Impact of procedure errors on ReDS

**Figure 3:**
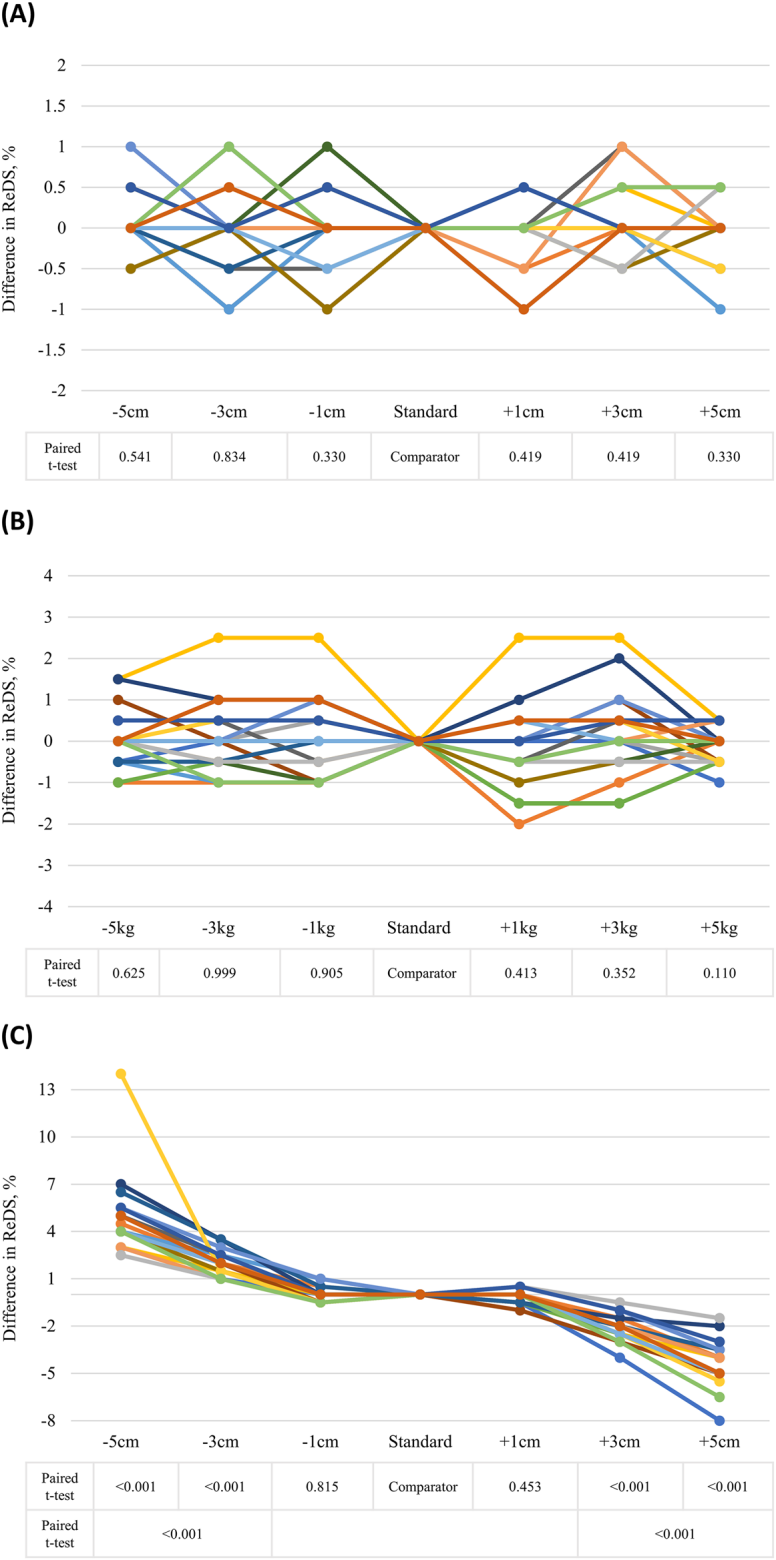
Impact of procedure errors (**A**) height, (**B**) weight, and (**C**) chest size on remote dielectric sensing (ReDS) measurements. Paired t-tests were conducted to compare each measurement with the standard measurement, unless stated otherwise.

**Figure 4:**
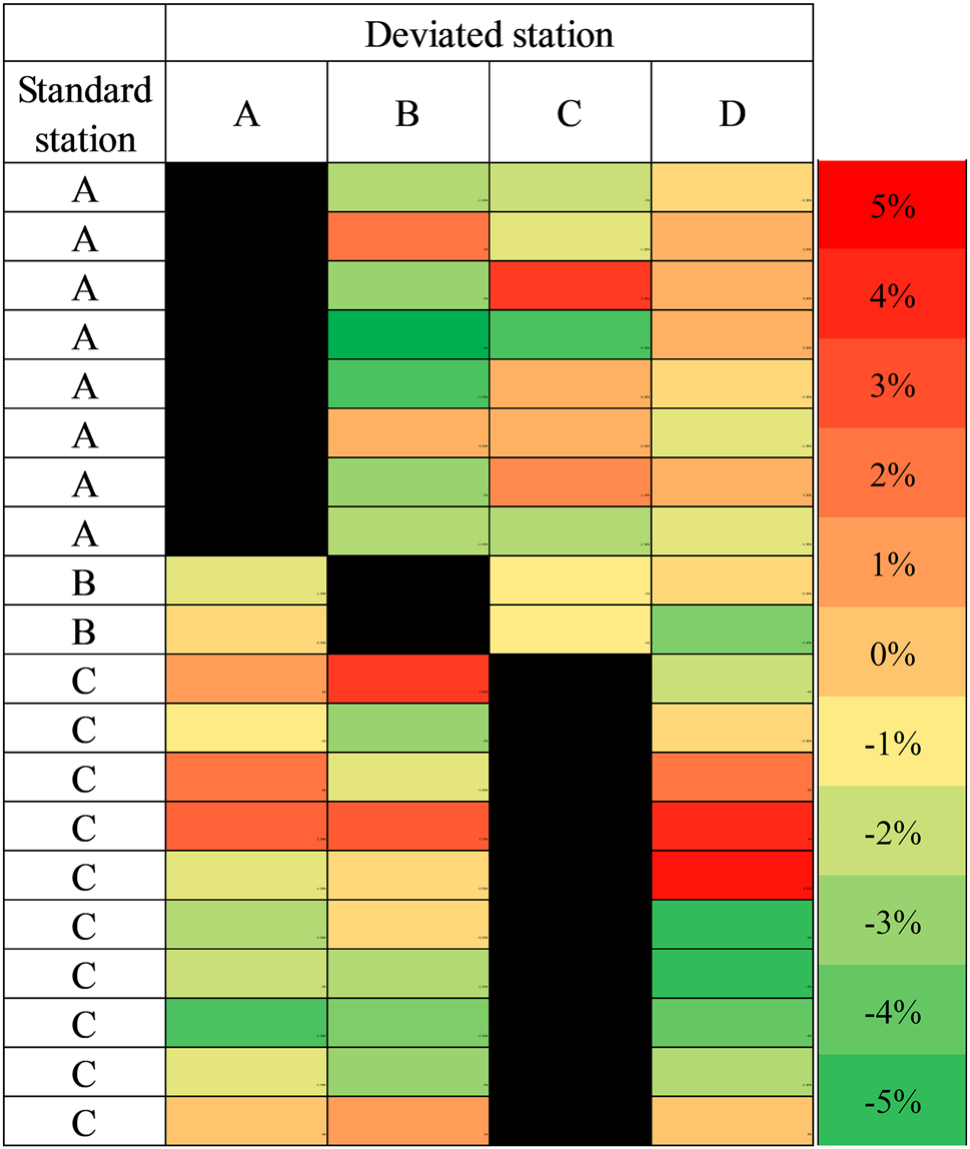
Effects of station switching on remote dielectric sensing (ReDS) measures. The different colors indicate the direction and magnitude of changes observed in the ReDS readings when the standard station was switched to other stations.

In the second part of the study, a total of 20 subjects participated, with an average age of 37 years, including 7 males. The participants had an average body mass index of 25 kg/m^2^. Height deviations within ± 5 cm and weight deviations within ± 5 kg did not significantly affect the ReDS readings (Fig. 3A,B, respectively). However, variations of ± 3 cm or ± 5 cm in chest size led to significant alterations in ReDS measurements by approximately ± 2% or ± 4–5%, respectively (Fig. 3C). Decreasing the chest size entry led to an overestimated ReDS value, while increasing the chest size entry resulted in an underestimated ReDS reading. Furthermore, when the standard station was changed to other stations, the ReDS readings deviated from the original values in an unpredictable manner in terms of both direction and magnitude (Fig. 4).

## Discussion

In this study, we assessed ReDS measurements in a healthy Asian population, focusing on intra-rater reliability and the impact of procedure errors on ReDS readings. The key findings of our study can be summarized as follows: (a) all healthy Asian volunteers demonstrated ReDS values within the suggested normal range of 20–35%; (b) the intra-rater reliability of ReDS measurements, performed by a single well-trained operator, was found to be excellent; (c) errors in height entry up to ± 5 cm or weight entry up to ± 5 kg did not have a significant impact on ReDS values; (d) deviations in chest size by ± 3 cm or more had a noticeable effect, i.e., approximately ± 2%, on ReDS measures; (e) incorrect station selection also resulted in notable changes in ReDS readings. Overall, our findings suggest that the normal range of the ReDS system is applicable to an Asian cohort, and it exhibits excellent intra-rater reliability. However, manufacturer’s instructions should be followed carefully during the ReDS measurements, as procedure errors such as deviation in chest size measurement and error in station selection can significantly impact the accuracy of ReDS readings.

A recent study conducted by Imamura et al.^14^ in Japan, along with ours in Taiwan, both involving healthy subjects spanning a wide age range, demonstrated that all ReDS values fell within the manufacturer-recommended normal range. These findings support the validity of ReDS measures in a population with smaller physical stature. Furthermore, Imamura et al.^10^ reported excellent intra-rater reliability of the ReDS system, with an ICC ranging from 0.966 to 0.988, which aligned with the ICC of 0.968 observed in our current study. These results suggest that ReDS readings are reproducible when performed by a single operator and indicate the feasibility of using one-time measurements in clinical practice.

In contrast, the study by Imamura et al.^10^ also revealed that the inter-rater reliability of ReDS measures, as indicated by the ICC, could be lower (0.683–0.866). This suggests only moderate reliability when different operators are involved. In addition, there was observed variability in median ReDS values of up to 5% between two operators. Notably, the study did not provide a specific rationale or explanation for the significant variation in ReDS measures observed among different operators, highlighting the need for further investigation to better understand and address this variability.

Our study revealed that even minor deviations in the operating procedure can lead to altered ReDS readings, which may contribute to the suboptimal inter-rater reliability observed. Notably, changes in chest size measurements were found to have a significant impact on ReDS values. While the specific computing algorithm employed by the ReDS system was not accessible to us, we observed that increasing or decreasing chest size entry resulted in underestimated or overestimated ReDS readings, respectively. These findings highlight the sensitivity of ReDS measurements to variations in chest size. We recommend recording chest size measures alongside ReDS readings to ensure accuracy and consistency in results.

Additionally, accurate station selection as recommended by the ReDS system is crucial for consistent ReDS measurements. Incorrect placement of the station can result in fluctuations in ReDS values. As ReDS is increasingly utilized in clinical practice^15^, our study reinforces the need to adhere to standardized operating procedures recommended by the manufacturer. This adherence ensures reliable and accurate ReDS measurements, thereby enhancing the accountability and applicability of ReDS in healthcare settings.

A few limitations of this study should be acknowledged and discussed. First, our study was conducted at a single center, which may affect the generalizability of the findings. However, the consistency of our results with previous research on ReDS values in healthy subjects and the intra-rater reliability of the ReDS system partially validates our findings^10,14^. Further studies in multiple centers and diverse populations are needed to confirm the generalizability of our results. Next, the impact of procedure errors on ReDS readings was observed in a population with relatively small stature. Therefore, it remains to be investigated how these errors may affect ReDS measurements in populations with different physical statures. Furthermore, due to study design limitations, we can only infer that height deviations within ± 5 cm and weight deviations within ± 5 kg did not significantly affect the ReDS readings. The impact of more pronounced weight or height errors on the measurements remains uncertain. As one of the pioneering studies in this area, our findings provide valuable insights that can encourage further research by other clinicians to explore this aspect in different populations and study designs. Finally, this study focused exclusively on a healthy cohort, which raises valid concerns about the generalizability of the findings to individuals with comorbidities, such as heart failure. However, we anticipate that the core principle of adhering to standardized operating procedures for ensuring accuracy and consistency in ReDS measurements remains applicable across diverse patient populations. Undoubtedly, further validation studies involving specific patient groups serve as the gold standard for addressing this question.

The ReDS system is increasingly being recognized for its role in heart failure patient management, and our study provides valuable and novel insights into its operation. Our findings highlight the significance of avoiding procedure errors, as they can inadvertently impact the results of ReDS measurements. It is essential to consider a few important factors when conducting these measurements. Accurate chest size measurement is crucial for obtaining reproducible ReDS values, and adherence to the manufacturer's instructions for correct station selection is paramount. By keeping these considerations in mind, the reliability and accuracy of ReDS measurements can be optimized.

## Data Availability

The data underlying this article will be shared on reasonable request to the corresponding author.
